# The Battersea Anti-Vivisection Hospital

**Published:** 1910-01-08

**Authors:** 


### The Battersea Anti-vivisection Hospital.

The three recent inquests upon patients of the Battersea Anti-vivisection Hospital have once again drawn attention to the faults in connection with the administration of this charity. It will be remembered that when the King's Fund finally allowed a grant to the institution, it did so only upon the understanding, expressed by members of the Council, that the Committee would mend its ways and definitely proceed to put its house in order. That recommendation was scorned and regarded as an insult by the Board of Management, and a great deal of nonsense was talked and published regarding the alleged injustice, partiality, and prejudice of the Council of King Edward's Hospital Fund in having so long refused recognition to the institution and in having finally coupled its grant with this unpardonable recommendation. The evidence before the coroner's court, given in the recent inquests, however, clearly shows that the caution of the Council was fully warranted. That evidence reveals a state of affairs existing at the Battersea General Hospital which is nothing less than disgraceful, which ought not to be tolerated, and which calls for the most unqualified condemnation. The faults, it is interesting to note, are administrative: it is the system, not the medical officer, that is to blame for these unfortunate incidents which have led to the disclosures regarding the daily work of this institution. We know of no hospital where the medical officer has so strenuous a task before him as he apparently has here; no hospital where the nurses act as assistant medical officers as they apparently do here, and no hospital where the patients are allowed for several hours during the day to depend, in case of a sudden emergency, upon the inherent charity of a neighbouring general practitioner, as they apparently do here. The allegation that want of funds is responsible for these administrative faults is unavailing as an excuse. An institution that can maintain so useless and expensive a toy as the Buisson apparatus for treating a disease that does not exist in these islands, can surely find means for securing the services of a duly qualified assistant to help the medical officer in his work.

It is clearly evident that the Battersea Hospital stands in need of thorough inspection and criticism by those competent to judge in institutional matters. As matters stand, the coroner's jury has censured the hospital administration—a verdict that will have the unqualified approval of every sensible person—and the reported evidence shows that there is urgent need for reform. The hospital is not, and can never be, a proper up-to-date institution worthy of the public confidence until and unless it puts itself into line with all other modern institutions, and depends for its support upon excellence of administration and management rather than upon the invidious advertisement of a sentimental fad. For these reasons we strongly urge the members of the Board of Management to adopt the advice given to them last year, to drop their prejudices and cleanse their house and to put it in thorough order before appealing to the public for support. Under the existing system a recurrence of such unfortunate incidents as those of last month is only to be expected, and with that possibility ever present, no patient will feel any desire to allow himself to be treated by the Battersea Anti-vivisection Hospital. On the other hand, a thorough reorganisation and reform of the institution will, we feel sure, be in the best interests of a charity that, whatever its faults may be, and whatever selfish motives underlie its foundation, may still be of great value to the local community.

